# C-arm-Guided Paravertebral Block for Surgical Anesthesia in a High-Risk Cardiac Patient Undergoing Percutaneous Nephrolithotomy

**DOI:** 10.7759/cureus.77746

**Published:** 2025-01-20

**Authors:** Chandra S Singh, Neha Mishra, Shivani Pathania, Shivam Rai

**Affiliations:** 1 Anesthesiology, Ganesh Shankar Vidyarthi Memorial Medical College, Kanpur, IND

**Keywords:** c-arm guidance, dilated cardiomyopathy, paravertebral block, percutaneous nephrolithotomy, regional anaesthesia

## Abstract

Percutaneous nephrolithotomy (PCNL) is an effective, minimally invasive procedure for removing large or complex renal stones. While general anesthesia is commonly used, it may lead to complications such as hemodynamic instability. This procedure typically involves accessing the kidney through a small incision in the back, where a nephroscope and other instruments are inserted to break up and remove the stones. General anesthesia is standard, but it can cause fluctuations in blood pressure, heart rate, and oxygen levels, which may complicate recovery. In this case, a 51-year-old male with dilated cardiomyopathy and bilateral staghorn renal stones underwent right-sided PCNL under C-arm-guided paravertebral block (PVB) at the T8, T10, and T12 levels. The procedure was successful, with no adverse events and excellent postoperative analgesia. PVB offers a safe alternative to general anesthesia in high-risk cardiac patients, minimizing complications and improving recovery.

## Introduction

Percutaneous nephrolithotomy (PCNL) is a minimally invasive procedure used to remove larger renal calculi (greater than 2 cm) and complex staghorn stones [1]. It is considered the gold standard for these cases, offering advantages over open surgery, including reduced morbidity and faster recovery. Despite its minimally invasive nature, adequate analgesia is crucial intraoperatively and postoperatively. Anesthesia techniques such as general anesthesia and regional anesthesia are commonly employed. However, general anesthesia has several drawbacks, including significant hemodynamic fluctuations, drug side effects, postoperative pain, and occasionally difficult airway management. In contrast, regional anesthesia offers benefits such as superior analgesic coverage, reduced postoperative pain, and the ability for patients to remain awake and cooperate during positioning [1].

A C-arm-guided paravertebral block (PVB) can be performed easily in the operating theatre where PCNL is conducted. The spread of dye in the paravertebral space can be visualized using the C-arm. After confirming the dye's spread, a local anesthetic drug can be deposited similarly. The local anesthetic will spread within the paravertebral space, providing sensory effects within 20 minutes. This type of regional anesthesia is advantageous for cardiac patients with low ejection fraction because it avoids the side effects associated with spinal and general anesthesia. Additionally, minimal fluid administration is required with this block, making it particularly beneficial for high-risk cardiac patients.

PVB is an attractive technique for analgesia in patients undergoing cardiac surgery. Its advantages include safety, efficacy, ease of insertion, and avoiding risks associated with thoracic epidural anesthesia.

Several regional anesthesia techniques, including subarachnoid block, PVB, and erector spinae block, are used for PCNL. Each technique has its advantages and limitations. Subarachnoid block can cause bilateral intercostal muscle paralysis and a reduction in functional residual capacity, which may hinder oxygenation in patients with low ejection fraction, leading to poor postoperative outcomes. Additionally, spinal anesthesia may induce sudden hypotension due to sympathetic blockade, which is particularly concerning in cardiac patients [2-4].

On the other hand, a PVB provides unilateral nerve blockade, avoiding these hemodynamic issues and preserving lower limb motor function, promoting early patient mobilization [5].

In patients with dilated cardiomyopathy, where the left ventricle is enlarged and weakened, anesthesia choices must be carefully considered. The key goals include avoiding myocardial depression, maintaining adequate preload, preventing increased afterload, and minimizing tachycardia, hypotension, and bradycardia.

Numerous studies have explored the use of regional anesthesia in PCNL, focusing on sensory doses of local anesthetic for postoperative pain management [6-9]. This case report discusses the anesthetic management of a high-risk cardiac patient, where the risks and benefits were carefully considered. A C-arm-guided PVB at three levels (T8, T10, and T12) was successfully performed, thereby avoiding the risks associated with general anesthesia and subarachnoid block.

Using a C-arm for PVB is advantageous, as it is commonly available in many operating theatres, particularly those where urological procedures such as PCNL are performed. Alternative imaging modalities, such as ultrasound, may be costly and are subject to strict regulations under the PCPNDT Act, making them less accessible in many settings. This case highlights the successful use of a C-arm-guided PVB in high-risk cardiac patients, demonstrating a safe alternative to traditional anesthesia approaches.

## Case presentation

A 51-year-old male with a history of dilated cardiomyopathy (ejection fraction: 25%) and bilateral staghorn renal stones was scheduled for right-sided PCNL. His medications included carvedilol (10 mg daily) and torsemide (Dytor) (10 mg daily). On examination, his vital signs were stable, with a heart rate (HR) of 90/min, blood pressure (BP) of 100/60 mm Hg, respiratory rate (RR) of 14/min, and oxygen saturation (SpO2) of 98% on room air. Systemic examination revealed bilateral clear lung fields with decreased air entry at the bases. A cardiovascular examination showed normal heart sounds with no murmurs.

**Table 1:** Laboratory investigations CBC: complete blood count, ECG: electrocardiogram, ECHO: echocardiogram, LVEF: left ventricular ejection fraction, N/A: not applicable

Investigation	Initial value	Reference range	Interpretation
CBC	-	-	Normal
Electrolytes	-	-	Normal
Serum creatinine	1.9 mg/dL	0.6-1.2 mg/dL	Elevated
ECG findings	Q waves in V1-V5	N/A	Abnormal
ECHO findings	LVEF 25%, mild mitral regurgitation	N/A	Decreased LVEF

Two IV lines were secured. The patient was premedicated with an antiemetic, inj. ondansetron 4 mg IV, and inj. pantoprazole 40 mg IV. To reduce anxiety, inj. midazolam 1 mg was administered slowly via IV.

**Figure 1:**
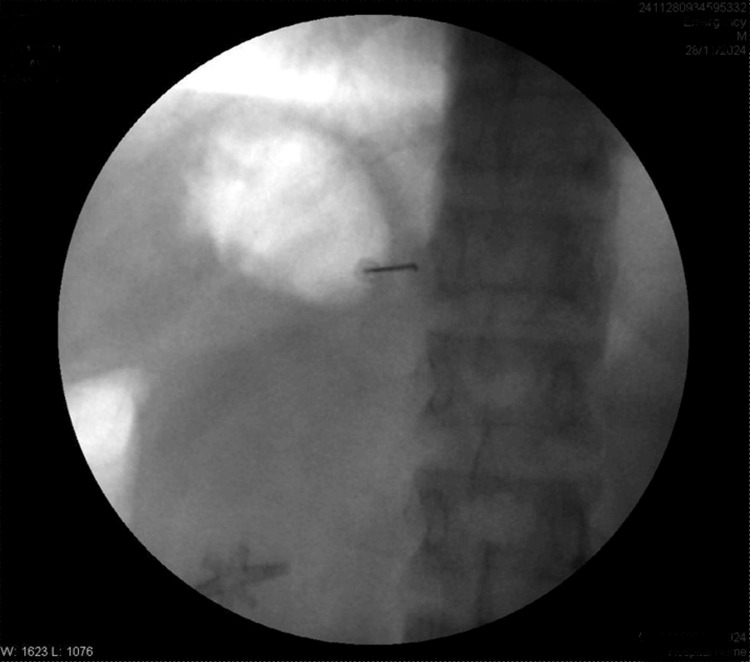
Needle at T12 transverse process

**Figure 2:**
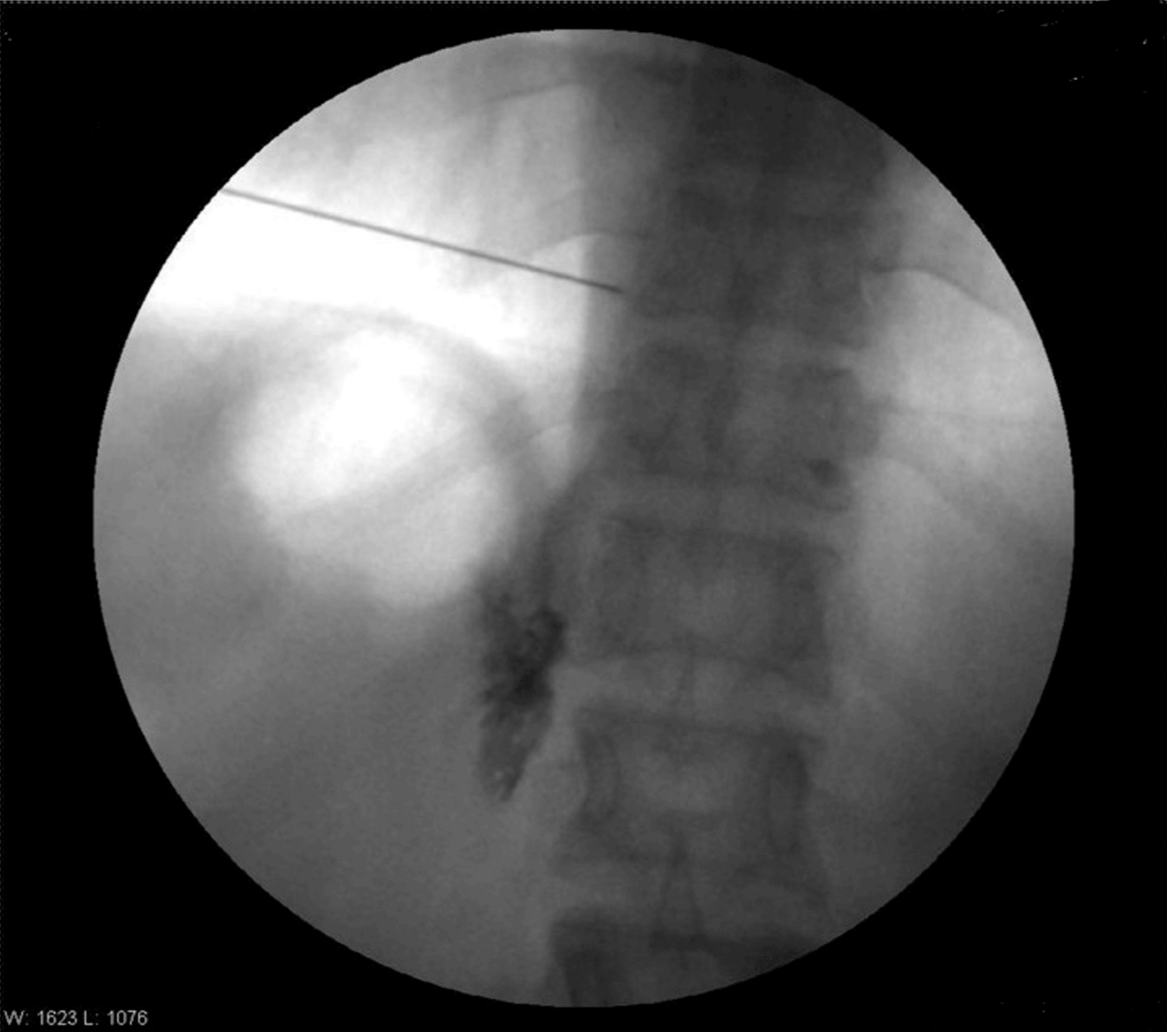
Anterior posterior fluoroscopic view after radio contrast dye iohexol 300 injection

**Figure 3:**
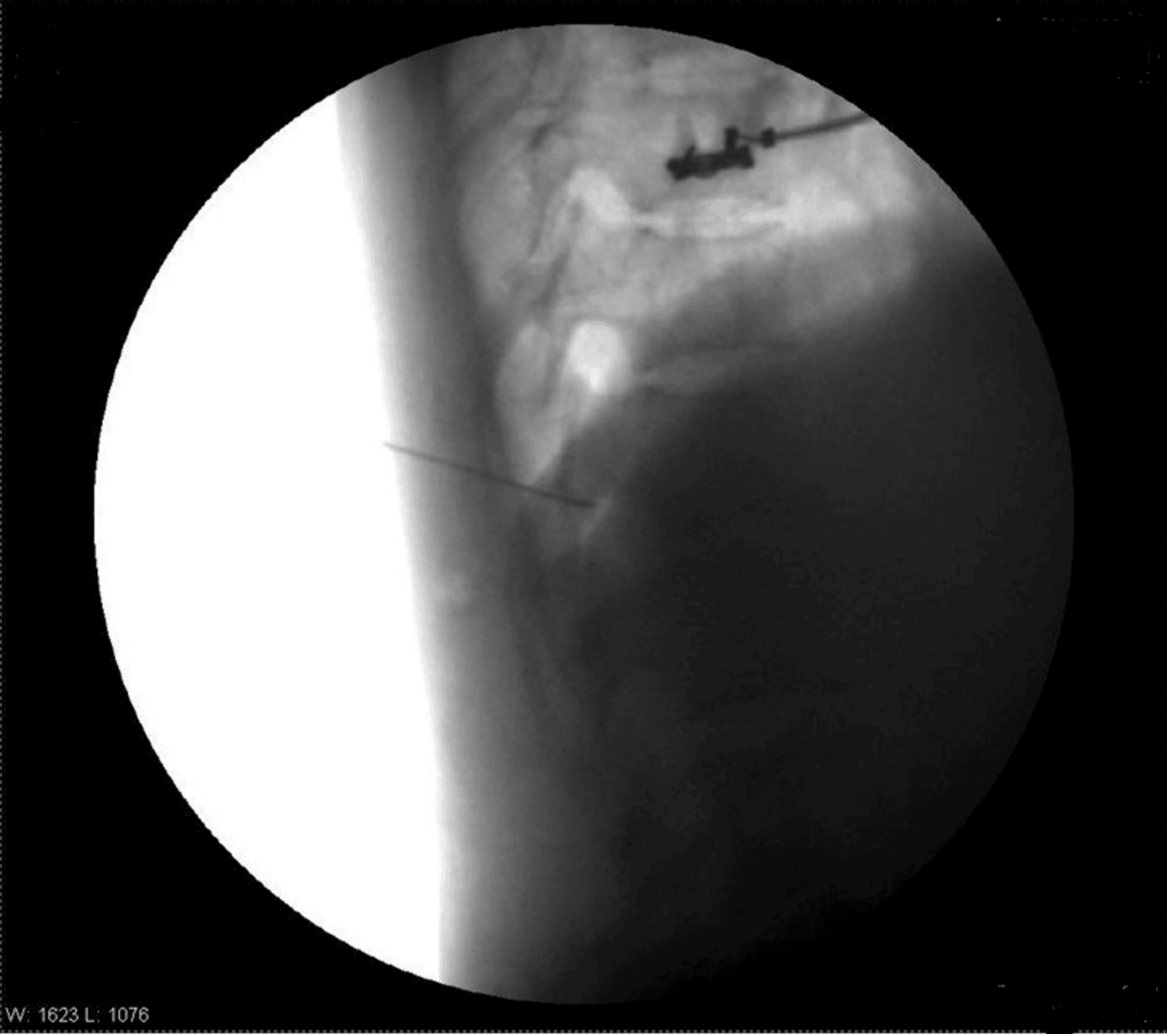
Needle at T12 lateral view

**Figure 4:**
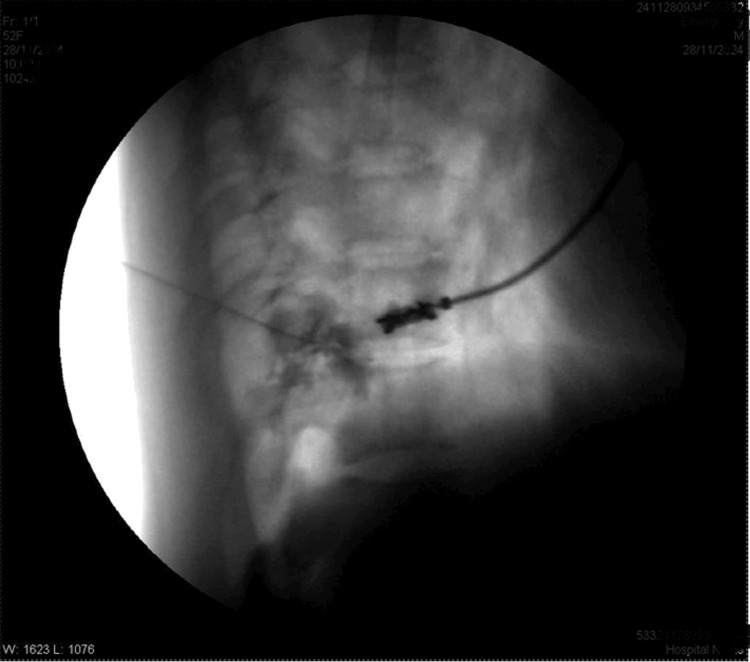
Lateral fluoroscopic view after radio contrast dye

The patient was positioned prone. After painting and draping, the transverse process of T8 was located on the right side with the help of a C-arm. A Quincke needle (22 G) was inserted under fluoroscopic guidance to reach the transverse process of T8. The needle tip was advanced caudally along the transverse process until a loss of resistance was felt. The needle's position was confirmed via fluoroscopy by injecting iohexol 300 radiocontrast dye, with images obtained in both anteroposterior and lateral views (Figures 1-4).

Four milliliters of levobupivacaine 0.5% were administered. Similar steps were performed for the T10 and T12 vertebrae. Sensory levels were assessed using hot and cold glass test tubes along the mid-axillary line. Motor blockade was evaluated using Snider’s match-blowing test, where the patient was asked to blow out a match held 15 cm from the mouth. Vitals after five minutes were HR of 90/min, BP of 100/68 mmHg, SpO2 of 98%, and RR of 14/min.

After this procedure, the patient was turned to a supine position. Under local anesthesia with lignocaine jelly and intravenous propofol at a dose of 0.5 mg/kg, cystoscopy was performed, and a guide wire was placed. Following confirmation of the sensory block from T8 to L2 and verification of motor blockade of the abdominal muscles using Snider’s match-blowing test, surgery was commenced.

The onset of sensory block was approximately 15 minutes. HR, BP, RR, and SpO2 were monitored every five minutes for the first 30 minutes and then every 10 minutes for the remainder of the surgery.

Parameters used to assess the patient status

Modified Bromage Scale

The Modified Bromage Scale is used to assess the motor block of the lower limb. A score of 0 indicates that the patient can lift their leg straight and move the hip, knee, and ankle. A score of 1 means the patient cannot lift their leg straight but can still flex the knee and ankle freely. A score of 2 shows that the patient cannot flex the knee or hip but can flex the ankle. A score of 3 indicates that the patient cannot flex the ankle, knee, or hip but can still move their toes. A score of 4 signifies no movement in the lower extremity.

Patient Satisfaction Scores

Scores were recorded on a scale of 0 to 5, with 1 point assigned for the absence of specific complaints such as postoperative pain, awareness during surgery, postoperative nausea/vomiting, postoperative urinary retention, or headache/backache.

Surgeon Satisfaction Scores

Scores on a scale of 0 to 5 were assessed based on the absence of pain during surgery, intraoperative movements, the need for sedation or conversion to general anesthesia (GA), postoperative side effects, and timely discharge from the hospital.

Visual Analogue Scale (VAS) Score

Scores were recorded on a scale of 0 to 10, where 0 represented no pain and 10 represented maximum pain.

The Bromage scale (used for assessing motor block in the lower limbs) was 2. The duration of the surgery was 1.5 hours. The patient did not complain of pain throughout the procedure. The surgery was completed successfully with minimal blood loss.

VAS scores (0 to 10, where 0 indicates no pain and 10 indicates maximum pain) were recorded intraoperatively. Our patient had a VAS score of 0. No adverse events, such as hypotension, hypertension, hypoxia, nausea, or vomiting, were noted. Both the patient and surgeon satisfaction scores were recorded and found to be satisfactory. In the postoperative period, the duration of analgesia was six hours.

## Discussion

In PCNL, a small incision is made in the flank using either a subcostal or supracostal approach. This incision allows for the location and fragmentation of renal stones using an energy source. At the end of the procedure, a ureteric stent and nephrostomy tube are placed. For PCNL, it is necessary to block skin and muscle innervation in the 10th and 11th intercostal spaces, as well as the visceral nerves of the kidney and ureter that originate from T10 to L1 [10]. Additionally, somatic pain from the incision site (T8-T12) must be addressed. A PVB given at T8, T10, and T12 can cover these dermatomes, making the surgery feasible.

Adequate pain control suppresses the surgical stress response and decreases the need for opioids. Regional anesthesia is considered a better technique for adequate pain management. PVBs are more beneficial than epidural or spinal anesthesia. The paravertebral space is a wedge-shaped area based on the lateral sides of the vertebral bodies and intervertebral foramina. The apex is continuous with the intercostal space. It is bounded posteriorly by the superior costotransverse ligament, anterolaterally by the pleura, medially by the vertebrae and intervertebral foramina, and superiorly and inferiorly by the ribs. Local anesthetic spreads in both cephalad and caudal directions, affecting the intercostal, interpleural, epidural, and prevertebral spaces [11]. A PVB produces fewer hemodynamic changes and adverse effects, such as nausea, vomiting, and hypotension, and it also reduces the length of stay in the hospital. Hemodynamic stability is achieved due to reduced sympathetic blockade and the unilateral, segmental nature of the block.

Prone positioning is required for accessing the upper urinary tract. Several complications are associated with prone positioning, such as in spinal anesthesia, where sudden hypotension can occur due to the compression of the great vessels, leading to decreased venous return. In general anesthesia, there is also a risk of accidental extubation. Increased intrathoracic pressure can also cause decreased left ventricular compliance, stroke volume, and cardiac output. This combination can result in a reduction in blood pressure, which may be detrimental in patients with dilated cardiomyopathy and low ejection fraction, potentially leading to arrhythmias and sudden cardiac arrest (low ejection fraction).

A PVB, which causes minimal hemodynamic changes in the prone position, is the most suitable technique for American Society of Anesthesiologists grade IV patients, avoiding the complications associated with spinal and general anesthesia. Moreover, a PVB provides a longer duration of analgesia, reducing the need for opioids [12]. Irrigation fluids are used in large quantities intraoperatively, so hypothermia must be prevented, as it can lead to postoperative complications such as delayed emergence and recovery from anesthesia, shivering, lactic acidosis, and excessive bleeding. Avoiding general and regional anesthesia is beneficial in such cases [13].

Our patient's surgery was successfully completed under a PVB with stable vitals. Moreover, we performed a cystoscopy after administering the PVB. For cystoscopy, the patient was turned supine, which provided us with the advantage of addressing any emergency situations and rescuing the airway if necessary. Euvolemia was maintained in our patient. Hypothermia was avoided using warm intravenous and irrigation fluids, and the patient was kept warm with a forced air warmer.

In their study, Elbealy et al. demonstrated that a lumbar PVB was superior to epidural anesthesia for PCNL, as indicated by lower pain scores and reduced use of systemic morphine [14]. Another study by Hatipoglu et al. concluded that in combination with IV tramadol in the postoperative period, ultrasound-guided PVBs with bupivacaine provide more effective analgesia than IV tramadol alone [15]. Baldea et al. conducted a study in which a PVB was performed at the level of T10 with a single injection of 20 mL of 0.5% bupivacaine [6,16]. The block was administered prior to surgery with the patient seated and under ultrasound guidance. The first dose of opioids for analgesia relief was given 119.7 minutes post-surgery in the PVB group.

A PVB is a good alternative for unilateral surgeries. It is devoid of side effects such as hypotension, urinary retention, and those associated with prone positioning during general anesthesia or subarachnoid block. Many studies have utilized USG-guided PVBs, especially for postoperative analgesia. However, our case report demonstrates a C-arm-guided PVB for surgical anesthesia during PCNL, not just for postoperative analgesia [17,18].

## Conclusions

The case highlights the significant benefits of C-arm-guided PVB as a safe and effective anesthetic technique for high-risk cardiac patients undergoing PCNL. PVB provides excellent analgesia, minimizes hemodynamic instability, and reduces the risk of cardiovascular complications, making it a valuable alternative to general anesthesia. Its ability to maintain stable blood pressure and heart rate, preserve motor function, and promote quicker recovery underscores its essential role in enhancing patient safety and improving postoperative outcomes in this vulnerable patient group.
